# Dynamic primitives in the control of locomotion

**DOI:** 10.3389/fncom.2013.00071

**Published:** 2013-06-21

**Authors:** Neville Hogan, Dagmar Sternad

**Affiliations:** ^1^Newman Laboratory for Biomechanics and Human Rehabilitation, Department of Mechanical Engineering, Brain and Cognitive Sciences, Massachusetts Institute of TechnologyCambridge, MA, USA; ^2^Department of Biology, Electrical and Computer Engineering, Physics, Center for Interdisciplinary Research on Complex Systems, Northeastern UniversityBoston, MA, USA

**Keywords:** discrete, submovement, rhythmic, oscillation, impedance, primitive, locomotion, rehabilitation

## Abstract

Humans achieve locomotor dexterity that far exceeds the capability of modern robots, yet this is achieved despite slower actuators, imprecise sensors, and vastly slower communication. We propose that this spectacular performance arises from encoding motor commands in terms of *dynamic* primitives. We propose three primitives as a foundation for a comprehensive theoretical framework that can embrace a wide range of upper- and lower-limb behaviors. Building on previous work that suggested discrete and rhythmic movements as elementary dynamic behaviors, we define submovements and oscillations: as discrete movements cannot be combined with sufficient flexibility, we argue that suitably-defined submovements are primitives. As the term “rhythmic” may be ambiguous, we define oscillations as the corresponding class of primitives. We further propose mechanical impedances as a third class of dynamic primitives, necessary for interaction with the physical environment. Combination of these three classes of primitive requires care. One approach is through a generalized *equivalent network*: a virtual trajectory composed of simultaneous and/or sequential submovements and/or oscillations that interacts with mechanical impedances to produce observable forces and motions. Reliable experimental identification of these dynamic primitives presents challenges: identification of mechanical impedances is exquisitely sensitive to assumptions about their dynamic structure; identification of submovements and oscillations is sensitive to their assumed form and to details of the algorithm used to extract them. Some methods to address these challenges are presented. Some implications of this theoretical framework for locomotor rehabilitation are considered.

## Introduction

In a recent publication, we asserted a pressing need for a fundamental mathematical theory to help organize and structure the prodigious volume of knowledge about sensorimotor control (Hogan and Sternad, 2012). We contend that such a theory has come within reach, though we anticipate that its development will require a process of continuous and incremental revision. While it is common practice to develop mathematical models for narrowly-specified sensorimotor tasks, to establish a reliable theoretical foundation it is necessary to take a broader perspective and consider the widest feasible range of behaviors—even if for no other reason than to uncover and confront facts that might prove embarrassing to a narrowly-formulated theory. Previously we outlined a theoretical framework for upper-extremity motor control that could encompass those quintessentially human behaviors, object manipulation and the use of tools. The goal of this essay is to extend this framework to lower-extremity motor control. To illustrate the potential value of such a theory we consider some of its possible implications for locomotor rehabilitation.

Of course, we acknowledge that an integrated theory of upper- and lower-extremity motor control is ambitious, but it ought to be possible—after all, there is only one central nervous system (CNS). Moreover, many commonplace actions require integrated control and coordination of upper and lower extremities, indeed of the entire body. For example, drilling a horizontal hole in a vertical wall using a hand-held drill is commonly performed in a standing position. Therefore, the force exerted by the hand on the drill and wall necessitates tangential force on the ground at the feet. In fact, almost all of the body's degrees of freedom must be coordinated—essentially everything between the hands and feet. The horizontal force results in an overturning moment that must be offset by displacing the center of gravity from the center of pressure below the feet, and a sufficiently strong hand force is typically accomplished by moving the center of gravity far beyond the base of support—i.e., by leaning hard into the push or pull (Dempster, 1958; Rancourt and Hogan, 2001). That is a common cause of falls if the horizontal force exceeds the frictional force between feet and ground and the feet slip (Grieve, 1983). Moreover, with feet together in this leaning posture, an unstable dynamic zero is introduced such that the hand force cannot *decrease* without transiently *increasing*, and vice-versa (Rancourt and Hogan, 2001). With feet far apart, that dynamic zero can be eliminated. The essential point is that the configuration of the *feet* dictates the dynamics of force exertion by the *hands*.

Even aside from the need to integrate upper- and lower-extremity motor control, the spectacular agility of human locomotion demands explanation. Even walking, that most mundane of behaviors, is a subtle and complex dynamic process. Despite intensive and ongoing research, the dynamics of human walking have yet to be reproduced by robots, even though they have actuators faster than muscle by factors of tens to thousands, and communication faster than neurons by a factor of a million or more (Kandel et al., 2000; Hogan and Sternad, 2012). But locomotor behavior is far more versatile than walking. For example, soccer, arguably the world's most popular sport, not only requires agile high-speed maneuvering to avoid equally agile opponents, but controlling the ball requires dexterity with the legs and feet comparable to that of the hands and fingers. In comparison, robot soccer—though fun, highly motivating, and a superb enticement to study science and engineering—is a pale shadow of the “beautiful game.”

## Dynamic primitives

Why is human locomotion so agile despite the limitations of our neuro-mechanical system? We believe that the answer lies in the distinctive character of human motor control. Mounting evidence indicates that sensorimotor control relies on a composition of primitive dynamic actions (Sternad et al., 2000; Thoroughman and Shadmehr, 2000; Flash and Hochner, 2005; Kargo and Giszter, 2008; Sternad, 2008; Sing et al., 2009; Degallier and Ijspeert, 2010; Dominici et al., 2011). We propose that *human motor control is encoded solely in terms of these primitive dynamic actions*.

Part of the challenge of controlling locomotor behavior is the high-dimensional, strongly nonlinear, hybrid character of the mechanical dynamics. “Hybrid” in this context refers to a mixture of discrete-event dynamics (each foot making or breaking contact with the ground changes the structure of the dynamic equations) and continuous dynamics (the motion of the skeleton in response to muscular action). With one foot on the ground, the human skeleton has on the order of 200 degrees of freedom; with two feet on the ground, a closed-chain kinematic constraint adds to the complexity. Moreover, kinematically-constrained rigid-body mechanics as described by Lagrange's equations is at best an approximation. Soft tissues contribute significantly to musculo-skeletal dynamics and add more degrees of freedom, e.g., via the deformation of muscles or body fat. For example, the impact due to heel strike can cause the mass of the calf muscles to resonate with the elasticity of passive tissues such as the Achilles tendon (Wakeling and Nigg, 2001; Wakeling et al., 2003). That phenomenon cannot be described by a model with only kinematically-constrained rigid bodies. The human body is a forbiddingly complex dynamic object. As we outline below, control via primitive dynamic actions may provide a way to manage this complexity.

The idea that motor control is accomplished by combining primitive elements is not at all new but the full extent of its ramifications for motor control may not yet have been fully articulated. The search for primitive elements that generate motor actions dates back at least a century. Sherrington proposed stereotyped neuromuscular responses to sensory events—the *reflexes—as* building blocks of more complex actions (Sherrington, 1906; Gallistel, 1980; Elliott et al., 2001). The subsequent wave of behaviorist psychology explored how stimulus-response associations (S-R units) could become an “alphabet” for complex behavior. Learning a new action would comprise “chaining” such S-R units or reflexes such that each reflexive action resulted in sensory events that “triggered” the next (Bässler, 1986).

Discrete and rhythmic movements have been proposed as candidates for two classes of primitive actions (Schaal et al., 2000, 2003; Sternad et al., 2000; Sternad, 2008; Ijspeert et al., 2013). They have been termed *dynamic* primitives as they refer to patterns of behavior that may robustly emerge from dynamic systems. To explain, two of the prominent behaviors exhibited by non-linear dynamic systems are *point attractors* and *limit cycles*; a point attractor may describe a discrete movement to a stable posture; a limit cycle may describe a rhythmic movement. Even some of the simplest dynamic systems can exhibit these behaviors as may be seen by considering the class of negative-resistance oscillators from engineering (Strauss, 1970). Those second-order dynamic systems can exhibit robustly sustained oscillation (limit cycle behavior) or stable convergence to a single state (point attractor behavior); changing the value of a single parameter is sufficient to select or induce a transition between these two alternatives. More biologically plausible models of neural oscillators exhibit similar properties, thereby lending support for these mechanisms generating observable behavior (Fitzhugh, 1961; Nagumo et al., 1962; Matsuoka, 1985; Ronsse et al., 2009).

Discrete and rhythmic movements describe unconstrained behavior but frequent physical contact with the ground is an inescapable aspect of human locomotion. A different class of dynamic primitives is required to manage that physical interaction. Locomotion is often described as “controlled falling,” yet most of the control occurs not during the fall, but during the sudden stop at its end. During single-legged support the body behaves approximately like an inverted pendulum and available control authority is quite limited. Most of the opportunities for control arise from the behavior of the swing leg as it contacts the ground. This dynamic “shock absorber” behavior is characterized by mechanical impedance. Controllable mechanical impedance is required as a third class of dynamic primitives to account for interaction, in locomotion as in object manipulation. We contend that, taken together, these three dynamic primitives may account for a wide range of behavior.

### Levels of analysis

To understand how dynamic primitives might account for human locomotor control, we distinguish between (at least) three levels of analysis: an *observational level* of overt, measurable behavior; a *combinatorial level* at which the dynamic primitives may be combined; and a *physiological level* from which the dynamic primitives may actually arise—e.g., through a combination of muscular and/or neural dynamics giving rise to submovements, oscillations, and impedances. These levels are loosely analogous to Marr's three levels of analysis—computational, algorithmic, and implementational (Marr, 1982). However, Marr's levels refer to computation or information-processing, specifically for vision. While control of locomotion also involves computation or information processing, the control of physical interaction is essential and not adequately subsumed under information processing.

A failure to distinguish between these levels—observational, combinatorial, and physiological—all too frequently confounds sensorimotor neuroscience. The definitions of dynamic primitives we propose below describe *product* rather than *process*. That is, in an attempt to establish a foundation, we focus on the phenomenology of motor behavior, not on specific hypothesized mechanisms that may give rise to that observable behavior. For clarity, we define dynamic primitives in the mechanical domain of motions and forces at the interface (points of contact) between the neuro-mechanical system and the physical world.

### Attractors

We define dynamic primitives as patterns of behavior that robustly emerge from dynamic systems, that is, as *attractors*. For example, a reasonably general representation of a dynamic system describes the evolution of behavior in a finite-dimensional state space, $\dot{x}=f(x)$ where **x** ∈ *R*^*n*^ for finite *n*. An attractor is a subset of state space with at least two properties: first, it is an *invariant set*: if the system begins in an invariant set, it never leaves it. Secondly, that invariant set is *attractive*: if the system starts sufficiently close to it, the system will ultimately converge to the attractor. Attractor sets may have many forms. A *point attractor* is a single point in state space. An attractor set that is a closed path (or orbit) defines a *limit cycle*. There are alternatives: any feasible path in state-space—any trajectory—may be an attractor; this may describe discrete reaching movements, which exhibit trajectory stability (Lackner and Dizio, 1994; Shadmehr and Mussa-Ivaldi, 1994; Won and Hogan, 1995; Burdet et al., 2001). Other subsets of state-space (e.g., manifolds) may also be attractors; these may describe synergies. Chaotic dynamic systems may have *strange attractors*, prodigiously complex objects with fractal geometry, and there is evidence that locomotion may exhibit chaotic dynamics (Strogatz, 1994; Hausdorff et al., 1995, 2001).

One important feature of this definition of dynamic primitives is that an attractor exhibits a degree of robustness that might be termed “*temporary permanence*”: permanence due to robustness to perturbation, temporary due to the fact that dynamic primitives may have limited duration. The pattern of behavior described by the invariant set will re-emerge after perturbation, at least for sufficiently small perturbations.

An important consequence of dynamic primitives defined as attractors is that it points to experiments that might test their objective reality (at least in principle). Due to the robustness of the attractor, a dynamic primitive should manifest as a common pattern of behavior observable in different contexts and despite the presence of noise or perturbations. This feature may lend itself to experimental testing.

### Discrete movements and submovements

An important requirement for a theory based on primitives is “composability”—it should be possible to combine the elements to generate a repertoire of behavior. In previous work we proposed precise quantitative definitions of discrete and rhythmic movements (Hogan and Sternad, 2007). Our definitions were deliberately confined to the behavioral or observational level, remaining silent about possible generative processes that might give rise to these observations. For a movement to be discrete, i.e., distinct from other movements, we reasoned that any consistent definition requires that it should begin and end with a period of no movement. With that definition, discrete movements can only be sequenced and cannot overlap in time. That would severely restrict the repertoire that could be generated.

To overcome this limitation, we propose that *submovements* are primitive dynamic elements of motor behavior. In essence, submovements are like discrete movements but they may overlap in time and their profiles may superimpose. A submovement is conceived as a *coordinative atom*: just as atoms are primitive units of chemical reactions, submovements are elements of dynamic coordination used to compose motor behavior. Just as atoms have complex internal structure, submovements may require complex patterns of neuromuscular activity to instantiate the dynamic process from which a submovement emerges as an attractor.

We define a submovement as an attractor that describes a smooth sigmoidal transition of a variable from one value to another with a stereotyped time-profile. For limb position, the variable is a vector in some coordinate frame **x** = [*x*_1_, *x*_2_… *x*_*n*_]^*t*^. If it is foot position in visually-relevant coordinates, the elements of **x** might be the positions and orientations of the foot (*n* ≤ 3 for location and *n* ≤ 6 if orientation is included). If it describes the configuration of the entire limb, the number of coordinates may be substantially greater, e.g., all of the relevant joint angles. Each coordinate's speed profile has the same shape which is non-zero for a finite duration *d* = *e* − *b*, where *b* is the time when the submovement begins and *e* is the time it ends, i.e., it has *finite support*:

$${\dot{x}}_{j}(t)={\hat{v}}_{j}\sigma (t),\;j=1\ldots n$$

where ${\hat{v}}_{j}$ is the peak speed of element *j*; σ(*t*) > 0 if *b* < *t* < *e* and σ(*t*) = 0 if *t* ≤ *b* or *e* ≤ *t*. The speed profile has only one peak: there is only one point *t*_*p*_ ∈ (*b*, *e*) at which $\dot{\sigma }(t_{p})=0$, and at that point, σ(*t*_*p*_) = 1.

Note that this definition is deliberately silent about possible generative dynamic processes that might give rise to submovements. However, some constraints on those processes can be identified. It may seem that a dynamic process with a point attractor is appropriate. However, physiological evidence shows that at least in reaching movements, the CNS does not simply specify final position (Bizzi et al., 1984; Won and Hogan, 1995). It is the trajectory, rather than final position, that is controlled. Further, this trajectory has a stereotyped time profile (Atkeson and Hollerbach, 1985). This dynamic primitive may be termed a “trajectory attractor.”

#### Composability

Submovements may be considered as *basis functions* and combined with overlap in time to produce a wide range of motion profiles. Though several combination operators are possible, linear vector superposition of discrete point-to-point reaching movements has been shown to provide an accurate description of movement trajectories in which a target shifts abruptly (Flash and Henis, 1991). Combining *m* submovements yields

$${\dot{x}}_{j}(t)=\sum_{k=1}^{m}{\hat{v}}_{jk}\sigma (t|b_{k},d_{k}),j=1\ldots n$$

where each submovement *k* has the same shape but may have different peak speed ${\hat{v}}_{jk}$, start time *b*_*k*_ and duration *d*_*k*_.

Composability has its drawbacks. One important disadvantage is the concomitant difficulty of identifying submovements unambiguously from a continuous motion record. Some responses to this challenge are discussed below.

### Rhythmic movements and oscillations

From a strictly mathematical perspective, a rhythmic dynamic primitive is not essential. Rhythmic movements could be described parsimoniously as a composite of overlapping submovements in opposite directions. However, rhythmic movement is very old phylogenetically. Available evidence indicates that oscillatory behavior of both upper and lower extremities is a *distinct* dynamic primitive element of biological motor control and not a composite of submovements (Brown, 1911, 1914; Grillner and Wallen, 1985; Schaal et al., 2004).

Because the term “rhythmic” has numerous confusing variations of meaning, to render precision, we denote the corresponding dynamic primitive as an *oscillation* (Hogan and Sternad, 2007). Describing limb position as a vector quantity, **x** = [*x*_1_, *x*_2_… *x*_*n*_]^*t*^, we define the primitive as an attractor that describes almost-periodic motion:

$$\left|x_{j}(t)-x_{j}(t+△t+lT)\right|<\varepsilon_{j}\;\forall t,\;l=\pm 0,1,2,\ldots ,\;j=1\;\ldots n,$$

where *T* is a constant (its smallest value is the period), |△*t*| < δ and ε_*j*_ and δ are small constants. This definition allows for the ubiquitous fluctuations exhibited in biological behavior, whether due to stochastic processes (noise) or deterministic chaos (Raftery et al., 2008). The main point of this definition is that the *average* time-course of an almost-periodic behavior is strictly periodic. The amplitude and phase of each vector component may differ but all components exhibit an average time-variation with the same shape and period, *T*.

As with submovements, this definition is deliberately silent about possible generative processes that might give rise to these observations. However, it seems reasonable to conjecture that oscillations emerge from a generative dynamic process with a limit cycle attractor (Kay et al., 1991; Rabinovich et al., 2006).

#### Composability

Evoking Fourier's theorem, it is clear that a wide range of almost-periodic behaviors may be composed by superposition of oscillatory primitives,

$${\bar{x}}_{j}(t)=\sum_{k=1}^{m}{\hat{x}}_{jk}\;s(t|T_{k},\phi_{k})$$

where the overbar denotes an average, *s*(*t*|*T*_*k*_, ϕ_*k*_) is a sinusoid with period *T*_*k*_ and phase ϕ_*k*_ as parameters, and ${\hat{x}}_{jk}$ is its amplitude. However, as with submovements, composability also implies a challenge. Unless their form is known precisely, unambiguous identification of type and number of oscillatory primitives from a continuous motion record is problematic.

### Mechanical impedance

To account for contact and physical interaction with the ground, a third class of dynamic primitives is required, mechanical impedances. Loosely speaking, mechanical impedance is a generalization of stiffness to encompass nonlinear dynamic behavior (Hogan, 1985). Mathematically, it is a *dynamic operator* that determines the force (time-history) evoked by an imposed displacement (time-history). The force and displacement must be *energetically conjugate*; that is, they must refer to the same point(s) so that incremental mechanical work *dW* may be defined i.e.,

$$dW=f^{t}dx=\sum_{j=1}^{n}f_{j}dx_{j}$$

where **x** = [*x*_1_, *x*_2_… *x*_*n*_]^*t*^ is a vector of positions and **f** = [*f*_1_, *f*_2_… *f*_*n*_]^*t*^ is a vector of forces, both defined with respect to any suitable coordinate frame. A mechanical impedance operator **Z** maps displacement onto the conjugate force.

f(t)=Z{△x(t)}

The form of this mapping may be nonlinear and time-varying. For convenience we often assume a state-determined representation

$$\begin{array}{l} \dot{z}=Z_{s}(z,△x,t)\\ f=Z_{o}(z,△x,t) \end{array}$$

where **z** = [*z*_1_, *z*_2_…]^*t*^ is a vector of state variables and *Z*_*s*_ and *Z*_o_ are algebraic functions. For brevity, we often omit the “mechanical” prefix.

The displacement inputs need not be at the same physical location in space, provided they can be paired with energetically-conjugate forces. For example, the several joints of the lower extremity (hip, knee, ankle, etc.) are in different physical locations. The limb configuration may be described using joint angles, θ = [θ_1_, θ_2_… θ_*n*_]^*t*^, a special case of *generalized coordinates* (Goldstein, 1980). The corresponding *generalized forces* (joint torques) τ = [τ_1_, τ_2_… τ_*n*_]^*t*^ are defined such that incremental mechanical work may be defined.

$$dW=\tau^{t}d\theta =\sum_{j=1}^{n}\tau_{j}d\theta_{j}$$

Joint mechanical impedance maps joint angular displacements onto the evoked joint torques.

$$\tau (t)=Z_{joint}\left\{△\theta (t)\right\}$$

Like submovements and oscillations, humans can voluntarily control mechanical impedance (Hogan, 1979, 1980, 1984, 1985; Burdet et al., 2001; Franklin and Milner, 2003; Franklin et al., 2007). The most obvious way is by co-contraction of antagonist muscle groups but the configuration of the limb also has a profound effect—posture modulates impedance (Hogan, 1990). During locomotion the mechanical impedance of the lower limb at the point of contact with the ground, and hence the way it absorbs or transmits the shock of impact to the rest of the body, depends strongly on whether first contact is made with the heel or the ball of the foot, or whether the leg is straight or the knee slightly flexed.

Mechanical impedance is a different kind of primitive than a submovement or oscillation; nevertheless it has properties of an attractor as we identified above. Mechanical impedance is extremely robust to contact and interaction. While the force and motion of the foot are obviously sensitive to contact with the ground, mechanical impedance at, say, the ankle is a property that emerges solely from the dynamics of the neuro-mechanical system supporting the foot and is completely independent of contact. It exhibits the robustness that we require for a dynamic primitive. Neuro-muscular mechanical impedance depends on the intrinsic physical properties of the muscular and skeletal systems but it is also influenced by neural feedback loops, especially those involving muscle spindles and Golgi tendon organs at the spinal level or higher. A compelling case has been made that one important function of these feedback loops is to maintain the mechanical impedance of the neuro-muscular actuator (Nichols and Houk, 1976; Hoffer and Andreassen, 1981). Undesirable impedance reduction due to cross-bridge detachment evoked by imposed displacement is corrected by enhanced neural activation; this makes the impedance an attractor of the closed-loop system. Moreover, it is known that the gains of these feedback pathways are highly modifiable, either via gamma motoneuron activity or via descending drive to spinal interneuron pools (Prochazka et al., 2000). Thus, the attraction to a particular impedance that these feedback loops provide has the *temporary permanence* that we believe is a hallmark of dynamic primitives.

#### Composability: superposition of impedances

A remarkable feature of mechanical impedance is that, when coupled to skeletal inertia, *non-linear impedances may be combined by linear superposition* (Hogan, 1985). That is, given a set of different impedances {**Z**_1_, **Z**_2_, … **Z**_*k*_} appropriate for different aspects of a task, the total impedance is

$$Z_{total}=\sum_{k=1}^{m}Z_{k}$$

even if any or all of the component impedances, **Z**_*k*_ are non-linear. These are among the reasons why modulating mechanical impedance is a particularly efficacious way to control interaction tasks (Toffin et al., 2003; Hogan and Buerger, 2004; Franklin et al., 2007). They are also the reasons why we believe that mechanical impedance is an essential dynamic primitive for contact tasks.

## Combining different classes of dynamic primitives

A theory based on dynamic primitives requires specification of how those primitives may be combined. An example may illustrate the challenge: a successful soccer kick requires skillful placement of the stance foot relative to the ball, a vigorous but carefully controlled motion of the swinging leg, and determination of appropriate impedance between foot and ball at the moment of contact, usually against the background of rhythmic running^1^. It is therefore essential to specify how the different dynamic primitives interact to produce observable forces and/or motions.

To do so we use the construct of a *virtual trajectory*, denoted **x**_0_. It summarizes the net motion due to commands from the CNS when the force exerted is identically zero. We make the mild assumption that the mechanical impedance is such that if the force is identically zero, the corresponding displacement is also identically zero: **f** ≡ 0 ⇒ △**x** ≡ 0 or in words, if force and all of its time derivatives and integrals are identically zero, then the corresponding displacement and all of its time derivatives and integrals are also identically zero. This allows us to *define* the displacement input to the impedance operator, △**x**, to be the difference between virtual (zero force) and actual (non-zero force) trajectories: △**x** = **x**_0_ − **x**, or in joint coordinates, △θ = θ_0_ − θ (Hogan, 1985). If the force is zero, the virtual and actual trajectories coincide. If the force is non-zero, the virtual trajectory **x**_0_(*t*) may be inferred from a knowledge of mechanical impedance **Z**, force **f**(*t*), and actual motion **x**(*t*) as **x**_0_(*t*) = **x**(*t*) + **Z**^−1^{**f**(*t*)}. This requires the inverse mapping **Z**^−1^{·} to exist. Note that the magnitude of impedance may be small provided it is non-zero.

This is not the only way these three classes of dynamic primitives—submovements, oscillations and impedances—might be combined, but an advantage of this construction is that it defines a non-linear extension of the *equivalent networks* widely used in engineering to describe physical interaction between dynamic systems, e.g., an audio amplifier and the speakers it drives (Hogan, 1985; Johnson, 2003a,b; Hogan, in revision). According to our view of dynamic primitives, the virtual trajectory **x**_0_(*t*) specified by the CNS may be composed of *submovements* and/or *oscillations*. Based on the difference between virtual and actual trajectories, *impedances* specified by the CNS determine the forces evoked by contact. With this representation, much prior engineering insight about dynamic interaction in machines may be re-purposed to help understand physical interaction in locomotion.

### Relation to the lambda hypothesis

The virtual trajectory is related to the “lambda” or “equilibrium-point” hypothesis but is also distinct from it in important ways. A common theme running through the several variants of the lambda hypothesis is the proposal that the CNS encodes motor commands as time-varying equilibrium postures (Feldman, 1966, 1986; Feldman and Latash, 2005). This is a proposed description of at least part of the process of generating movement. However, the mere existence of an “instantaneous equilibrium point,” though not guaranteed, is not by itself very surprising from a physiological perspective; for example, the variation of muscle tension with length may suffice. Therefore, an instantaneous equilibrium point does not by itself provide compelling evidence about how the CNS encodes motor commands.

To define a virtual trajectory only requires that mechanical impedance—a physically measurable quantity—has a well-defined zero as described above. Most descriptions of the neuromuscular actuator satisfy this requirement. If so, an “instantaneous equilibrium point” (which we term a virtual position) may always be defined. This construct is a consequence of observable neuro-muscular mechanics. It is a description of behavior (the *product*) and may have no relation to how the CNS goes about producing that behavior (the *process*). Existence of a virtual trajectory does not require or imply that the CNS knows about or uses this construct for control. Indeed, available evidence suggests that this would be at best an incomplete account (Lackner and Dizio, 1994).

### Dynamic primitives in locomotion

What is the evidence that these dynamic primitives describe the control of locomotion? Walking clearly exhibits a strongly rhythmic character, but that by itself is not sufficiently informative; walking could be a sequence of discrete (or overlapping) steps and in some cases—e.g., the slow pacing used in a funeral march—it may be. Furthermore, what is the role of mechanical impedance?

#### Role of oscillations and submovements

Observations of fictive locomotion in non-human vertebrates provide unequivocal evidence that neural circuits capable of generating an oscillatory dynamic primitive—sustained rhythmic activity—exist in the spinal cord isolated from its periphery, though sensory feedback is known to play a key role (Brown, 1911; Grillner and Wallen, 1985; Kriellaars et al., 1994; Stein et al., 1995; Cazalets et al., 1996; Grillner et al., 1998; Pearson et al., 2004). For unimpaired humans, continuous leg muscle vibration produced locomotor-like stepping movements, and spinal electromagnetic stimulation applied to unimpaired human vertebrae induced involuntary locomotor-like movements (Gurfinkel et al., 1998; Gerasimenko et al., 2010). That suggests the existence of a rhythmic central pattern generator (CPG) in the human spinal cord that may contribute to generating locomotor activity, though feedback elicited by limb loading, hip extension or the pressure on the sole the foot also play important roles (Grillner and Wallen, 1985; van Wezel et al., 1997; Dietz and Harkema, 2004).

However, the relative contribution of rhythmic pattern generation to unimpaired human locomotion remains unclear. Human infants exhibit a primitive rhythmic stepping reflex but it typically disappears at about 6 weeks after birth without training (Yang and Gorassini, 2006). When independent walking emerges at about one year old, it does not initially exhibit the rhythmic pattern of mature walking and this cannot be ascribed to immature postural control (Ivanenko et al., 2005). Furthermore, the locomotor-like movements evoked by stimuli to unimpaired human subjects were observed in a gravity-neutral position, unlike normal walking, rendering it difficult to assess how those results would apply to upright walking (Gurfinkel et al., 1998; Gerasimenko et al., 2010).

Walking in unimpaired adults is characterized by a remarkably repeatable spatial trajectory of the foot (Ivanenko et al., 2002). In response to surface irregularity in the form of small obstacles, subjects adjusted their minimum toe clearance using subtle adjustments of lower-limb kinematics (Schulz, 2011). Patients with spinal cord injury (SCI) who recovered following body-weight supported treadmill training generated a foot trajectory that closely matched the normal pattern, although they used very different joint coordination patterns to do so (Grasso et al., 2004). Together, these observations suggest the presence of a trajectory attractor underlying foot motion similar to that underlying hand motion in simple reaching movements (Bizzi et al., 1984; Lackner and Dizio, 1994; Shadmehr and Mussa-Ivaldi, 1994; Won and Hogan, 1995; Burdet et al., 2001).

Given our definition of dynamic primitives as attractors, studying the stability properties of ambulatory behavior may help resolve this question. Robustly sustained oscillation emerges as a *limit cycle attractor* from nonlinear dynamical systems such as relaxation oscillators (van der Pol, 1926). Nonlinear limit cycle oscillators not only encapsulate the robust and stable rhythmic motion of the periphery in human walking; they also serve as competent models of neural rhythmic pattern generators (Matsuoka, 1987; Taga et al., 1991; Collins and Richmond, 1994; Taga, 1998; Rybak et al., 2006). One of their distinctive characteristics is that they may exhibit *dynamic entrainment*: under certain conditions they will synchronize their period of oscillation to that of an imposed oscillation and *phase-lock* to establish a particular phase relation with it (Bennett et al., 2002). Usually entrainment occurs only for a limited range of frequencies; it exhibits a *narrow basin of entrainment*. In fact, entrainment to periodic mechanical perturbation has been reported in several non-human vertebrates which show clear evidence of spinal pattern generators (Grillner et al., 1981; Pearson et al., 1992; McClellan and Jang, 1993; Kriellaars et al., 1994).

**Figure 1:**
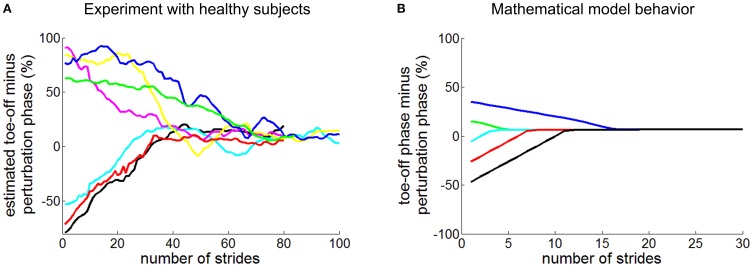
**Entrainment of human walking to periodic ankle torque pulses**. Panel **(A)** shows experimental observation of unimpaired subjects (Ahn and Hogan, 2012b). The phase difference between toe-off (initiation of swing) and the initiation of the perturbation pulse is plotted as a function of stride number. Panel **(B)** shows comparable data generated by a simple mathematical model for various initial phases of the perturbation pulse (Ahn and Hogan, 2012a). In both **(A** and **B)**, regardless of initial phase, the perturbation pulses converged to phase-lock at the end of double stance, close to toe-off.

A recent study reported behavioral evidence that a neuro-mechanical oscillator contributes to human walking, though perhaps weakly (Ahn and Hogan, 2012b). As unimpaired human subjects walked on a treadmill at their preferred speed and cadence, periodic torque pulses were applied to the ankle. Though the torque pulse periods were different from their preferred cadence, the gait period of 18 of 19 subjects converged to match that of the perturbation (Figure 1A). Significantly, this entrainment occurred only if the perturbation period was close to subjects' preferred walking cadence: it exhibited a narrow basin of entrainment. Further, regardless of the phase within the walking cycle at which the perturbation was initiated, subjects' gait synchronized or phase-locked with the mechanical perturbation at a phase of gait where it assisted propulsion. These results were affected neither by auditory feedback nor by a distractor task. However, the convergence to phase-locking was slow, requiring many tens of strides.

The existence of a basin of entrainment, however narrow, indicates that a non-linear limit-cycle attractor underlies level treadmill walking but it does not discriminate between several physiologically-plausible mechanisms that might be responsible, e.g., a CPG in the spinal cord or a “closed chain” of reflexive actions such that each results in sensory events that “trigger” the next (Bässler, 1986; Gurfinkel et al., 1998; Gerasimenko et al., 2010). Nevertheless, a highly-simplified mathematical model in which afferent feedback triggered actuation of the trailing leg reproduced all of the features observed experimentally (Figure 1B): (1) a periodic bipedal walking pattern; (2) local asymptotic stability of that periodic walking pattern; (3) entrainment of that walking pattern to periodic mechanical perturbations with a narrow basin of entrainment; and (4) phase locking to locate the perturbation at the end of double stance when entrained (Ahn and Hogan, 2012a; Ahn et al., 2012).

#### Role of musculo-skeletal mechanical impedance

A key insight derived from that model is that stable locomotion requires energy dissipation. Although collision-free legged locomotion is physically possible, to the best of our knowledge non-elastic interaction between foot and ground, which dissipates kinetic energy, is a common characteristic of legged animal locomotion. In human locomotion, muscles do more positive than negative work, even when walking at constant average speed on level ground, which provides evidence of energy dissipation (Devita et al., 2007).

The impact between the foot and the ground happens very rapidly; foot-ground forces have significant frequency content up to 15 or 20 Hz and beyond (Antonsson and Mann, 1985; Wakeling and Nigg, 2001). The bandwidth of lower-limb muscles in response to neural excitation is no more than a couple of Hz and the shortest transmission delay associated with spinal feedback is 50 ms or longer. As a result, reactive control of foot-ground interaction based on neural feedback is unworkable. However, musculo-skeletal mechanical impedance enables controlled reactions much faster than neural responses. Modulating shock absorption and energy dissipation depends on pre-tuning lower-limb mechanical impedance, i.e., using impedance as a dynamic primitive of motor control. The magnitude of the required shock absorption varies with walking speed and variation of lower-limb stiffness with speed of human locomotion has been widely reported (Farley and Gonzalez, 1996; Ferris et al., 1999; Holt et al., 2003).

In the simplified mathematical model described above, ankle mechanical impedance also affected the energy added during the push-off phase; a pre-stretched spring-like muscle was released (Ahn and Hogan, 2012a; Ahn et al., 2012). Though this is at best a crude approximation to the action of lower-limb muscles, it yielded a more stable walking cycle (i.e., a larger basin of attraction) than simply modeling muscle action as generating a force or torque pulse with zero impedance. This further supports our contention that musculo-skeletal mechanical impedance is one of the essential dynamic primitives required for human locomotion.

#### Interaction between dynamic primitives in locomotion

If dynamic primitives underlie locomotion, then interaction between them may also play an important role. One mathematical simulation study suggested that a hybrid dynamic walker was more stable when synchronized with an oscillator that acted as a clock than when it operated independently (Seipel and Holmes, 2007). Notably, the interaction between the oscillator and the periphery was exactly analogous to the equivalent network we propose: the oscillator specified a nominal limb trajectory; joint torque was exerted, determined by a simple mechanical impedance, as a function of the difference between nominal and actual limb trajectories.

In addition to rhythmic cycling of the limbs, functional locomotion requires the ability to place a foot, e.g., to avoid an obstacle or to secure an appropriate foothold on the first step of a flight of stairs. This requires the production of a discrete step against the background of an ongoing rhythm. In principle, that might be achieved by simple linear superimposition of a virtual trajectory corresponding to a submovement onto one corresponding to an oscillation. However, upper-extremity studies have shown that, against the background of rhythmic motion, the onset of a discrete action preferentially occurs at selected phases of the ongoing rhythm, which implies a nonlinear interaction (Sternad et al., 2002). A model comprising a Matsuoka oscillator coupled to antagonist muscles acting about a single joint successfully reproduced this phenomenon (De Rugy and Sternad, 2003; Ronsse et al., 2009).

Whether similar phenomena occur in human walking is, to the best of our knowledge, unknown at this time. It seems clear that a single interposed discrete step—e.g., a sidestep—does not catastrophically disrupt an ongoing walking rhythm. However, it is less clear which aspects of that rhythm exhibit the stability of an attractor. Subjects exhibit a preferred cadence and step length that appears to be robust (MacDougall and Moore, 2005). However, transient lower-limb perturbations induce phase-resetting of the walking rhythm, a persistent change of phase relative to the pre-perturbation oscillation (Nomura et al., 2009; Feldman et al., 2011). This indicates that the oscillatory lower-limb trajectory, e.g., time history of joint angles, is not an attractor. Whether interposed side-steps evoke similar phase-resetting is a matter for future investigation.

### Identifying dynamic primitives in locomotion

**Figure 2:**
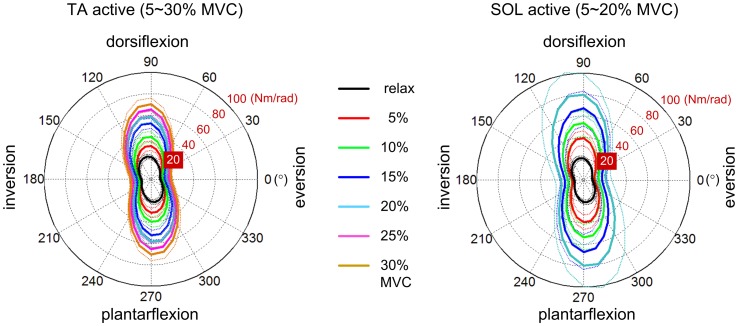
**Variation of static ankle mechanical impedance with muscle activation (Lee et al., 2012c)**. Polar plots show magnitude of restoring torque evoked by displacement in different directions. **Left panel**: Tibialis anterior active. **Right panel**: Soleus active. MVC denotes maximum voluntary contraction. Solid lines: mean of all subjects. Dotted lines: mean ± standard error. Note the pronounced weakness in inversion-eversion, the direction of most ankle injuries.

Some progress towards identifying dynamic primitives and their interaction in locomotion has been made. Experimental identification of impedance requires mechanical perturbation; the evoked response at the point(s) of interaction is determined by impedance. However, even the static component of multivariable joint impedance (the relation between torque and angular displacement) may be highly structured. Measurements on unimpaired subjects show a pronounced weakness in inversion-eversion, the direction of most ankle injuries (Figure 2). Increasing muscle activation increases stiffness but does not eliminate this relative weakness (Lee et al., 2012c).

**Figure 3:**
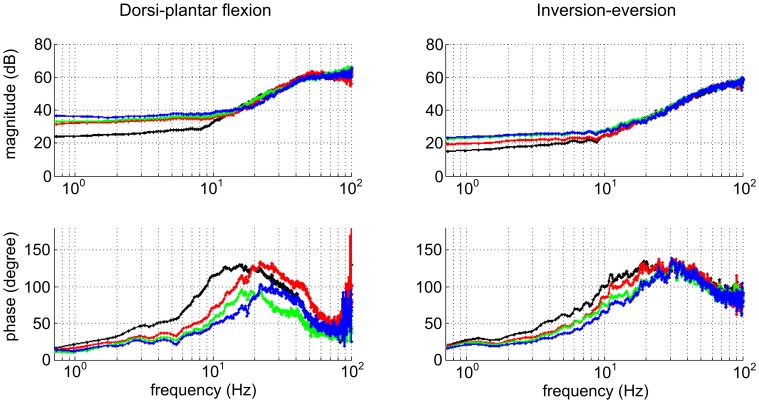
**Variation of dynamic ankle mechanical impedance with muscle activation (Lee et al., 2012b)**. Bode plots show impedance up to 50 Hz in principal axis directions. **Left column**: Dorsi-plantar flexion. **Right column**: Inversion-eversion. **Top row:** magnitude. **Bottom row:** phase. Color code: black, fully relaxed; red, tibialis anterior active at 10% maximum voluntary contraction (MVC); green, soleus active at 10% MVC; blue, tibialis anterior and soleus co-contracted, each at 10% MVC. The effect of muscle activation is to increase impedance below about 10 Hz, predominantly in dorsi-plantar flexion. The phase plots suggest dynamic behavior more complex than second-order.

It is common to assume that the combination of skeletal inertia and neuro-muscular impedance exhibits second-order dynamics (Dolan et al., 1993; Tsuji et al., 1995). Though that may seem reasonable, it is not necessarily correct and there is good reason to expect higher-order dynamic behavior (Wakeling and Nigg, 2001). To avoid *a-priori* assumptions about the order of the dynamics, stochastic methods may be used to identify a locally linear approximation to dynamic behavior (Palazzolo et al., 2007; Chang et al., 2012). They have been applied successfully (Figure 3) to identify the steady-state multi-variable dynamic impedance of the ankle (Rastgaar et al., 2009, 2010; Lee et al., 2012b).

**Figure 4:**
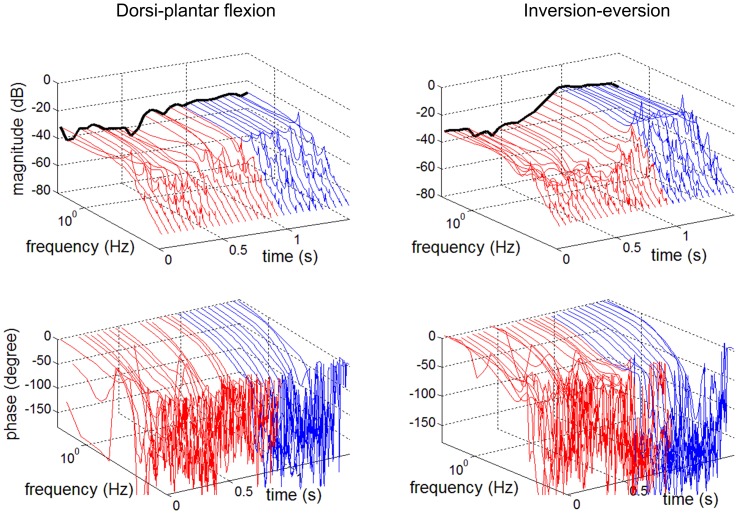
**Time-variation of ankle dynamics during level walking (Lee et al., 2012a)**. Bode plots of admittance (inverse of impedance) at 50 ms intervals. **Left column**: Dorsi-plantar flexion. **Right column**: Inversion-eversion. Top row: magnitude. Bottom row: phase. Time zero is at heel strike. Color code: red, stance phase; blue, swing phase. The black line depicts the static (zero frequency) component. Note the substantial magnitude changes throughout the gait cycle.

Stochastic methods may also be extended to identify *time-varying* mechanical impedance (Lortie and Kearney, 2001). They have recently been applied (Figure 4) to identify a time-varying trajectory of multivariable ankle mechanical impedance during level walking (Lee et al., 2012a; Lee and Hogan, 2013).

A virtual trajectory, **x**_0_(*t*), can also be measured experimentally. If the point of interaction is the sole of the foot, then during swing phase the force is identically zero, **f** ≡ 0, and because △**x** ≡ 0, the observed motion is the virtual trajectory. During stance phase the force is non-zero, f≡0 but **x**_0_(*t*) may be inferred from a measurement of mechanical impedance, **Z**, force, **f**(*t*), and actual motion, **x**(*t*) as **x**_0_(*t*) = **x**(*t*) + **Z**^−1^{**f**(*t*)} provided **Z**^−1^ exists. If the point of interaction is at a joint—say, the ankle or the knee—then the dynamics between the joint and the point of force application must be identified and subtracted. The main difficulty is that estimates are exquisitely sensitive to the *assumed* order of the neuro-muscular impedance model used to infer a virtual trajectory—see Gomi and Kawato (1996) but compare with Gribble et al. (1998). However, there is no fundamental reason it cannot be determined and model-independent experimental methods have been demonstrated (Hodgson and Hogan, 2000).

**Figure 5:**
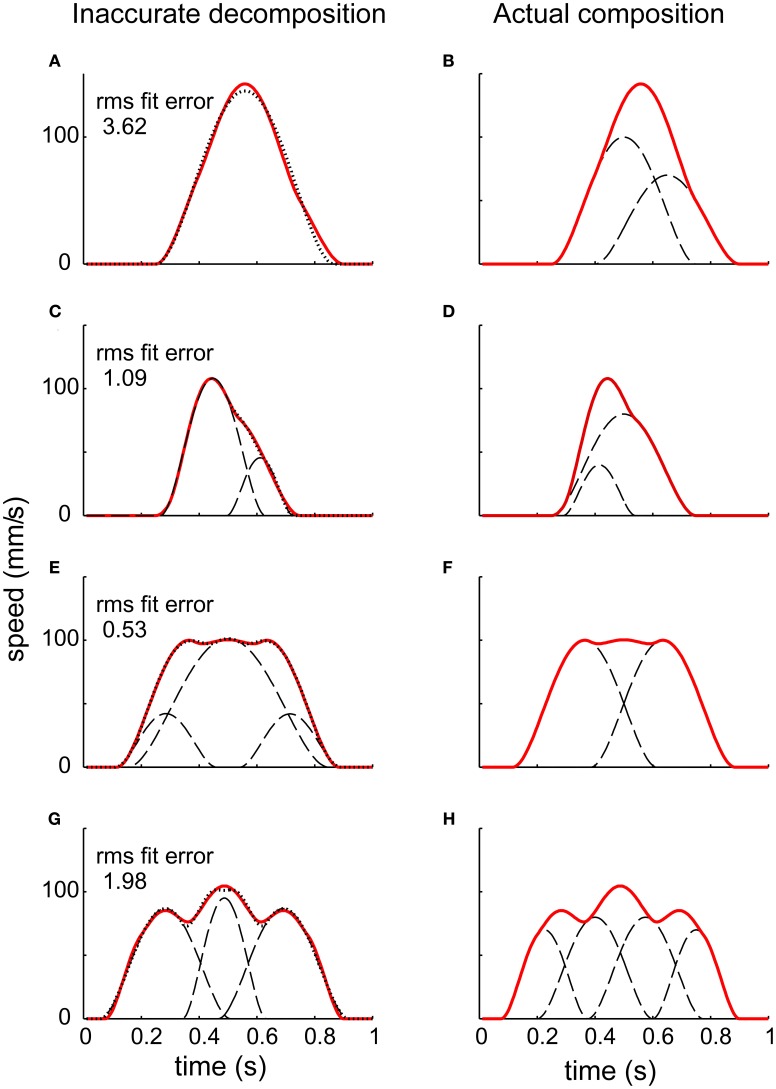
**The challenge of decomposing a continuous trajectory into submovements (Rohrer and Hogan, 2003)**. The right column shows simulated speed profiles resulting from different combinations of underlying submovements. Note that the number of peaks does not correspond to the number of submovements. The left column shows the result of decomposition using “greedy” algorithms. Though the RMS fitting error is low, the submovements identified do not resemble those used to construct the speed profiles.

Given a measured virtual trajectory, there remains the challenge of identifying underlying motion primitives, such as submovements and oscillations. Composability, the requirement that dynamic primitives may be combined to produce behavior, may introduce ambiguities. One common approach to identifying submovements is to examine derivatives of the trajectory to identify local peaks, but that method is completely unreliable (Figure 5). A composite of two smooth submovements may yield one, two, or three local velocity peaks (Rohrer and Hogan, 2003).

Alternative methods use “greedy” algorithms which first find a submovement that best fits the trajectory in some suitable sense (least residual error, highest peak speed, etc.), then subtract it and repeat the procedure on the residual until the error between the sum of submovements and the original trajectory falls below a specified threshold. Unfortunately, these methods also yield spurious decompositions (Figure 5). Even in a simulated “test” case, where a sequence of submovements is known *a-priori* and used to compose a continuous trajectory, these methods cannot reliably recover the underlying submovements (Rohrer and Hogan, 2003).

**Figure 6:**
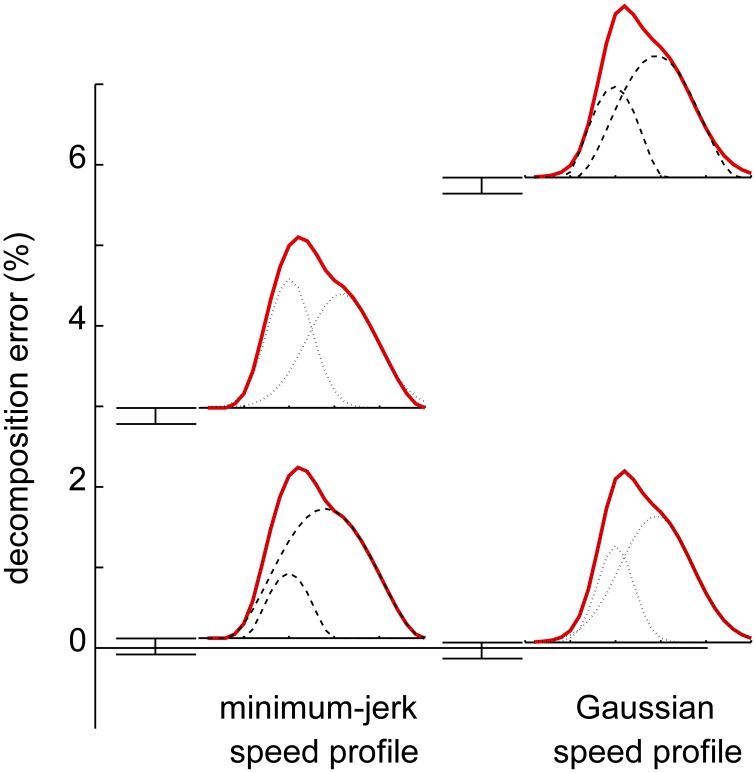
**Ability of decomposition based on global optimization to discriminate different submovement shapes underlying a speed profile (Rohrer and Hogan, 2003)**. Solid lines: simulated speed profiles. Dotted lines: Gaussian submovements. Dashed lines: minimum-jerk submovements. A speed profile composed of Gaussian submovements yields substantially greater fitting error when decomposed into minimum-jerk submovements (right column) and vice-versa (left column).

However, global optimization methods have been developed which avoid spurious decompositions (Figure 6). With these methods it has been shown that (1) the statistics of the extracted submovement parameters are robust to the assumed submovement shape and (2) the errors introduced by inappropriate submovement shapes can be detected even in the presence of substantial measurement noise (Rohrer and Hogan, 2003, 2006).

## Locomotor rehabilitation

A theoretical framework based on dynamic primitives may have particular relevance for sensorimotor rehabilitation, both in the development of assistive technologies and in the design of therapeutic procedures.

### Assistive technologies

The design and implementation of assistive orthoses and amputation prostheses has unequivocally demonstrated the importance of controllable mechanical impedance. It is a key element of recent highly-successful designs of ankle-foot orthoses and transfemoral prostheses (Blaya and Herr, 2004; Au et al., 2007; Sup et al., 2008, 2009; Ha et al., 2011; Lawson et al., 2011; Sup et al., 2011). A central feature of these designs is the equivalent network structure referred to above, which is used to combine neural and mechanical influences on how the foot interacts with the ground. However, it is less clear whether submovements and/or oscillations play a prominent role. For example, the designs by Goldfarb and colleagues implement a finite number of states arranged in a closed cycle (Sup et al., 2008). Rhythmic behavior emerges as consequence of this closed cycle rather than due to any neurally-generated oscillation. To anticipate future work, this new technology may provide essential tools to test a theory based on dynamic primitives.

### Physiotherapy

A theoretical framework based on dynamic primitives may also have a substantial value for therapies to *recover* neuro-motor function rather than assist it or replace it. To date, therapeutic practices have lacked a basis in experimentally-verified theory. This is understandable because there is, as yet, little scientific consensus on the neural control of unimpaired locomotion, and certainly none on how the CNS responds to injury. Nevertheless, it is difficult to understand how rational design of therapeutic procedures might be accomplished without a fundamental theory of locomotion and its recovery.

Most rehabilitation practices tacitly assume that motor recovery is loosely analogous to unimpaired motor learning. However, unimpaired motor learning happens in an intact nervous system and is not accompanied by the common sequelae of neurological injury, which include muscular weakness, spasticity, abnormal muscle tone, abnormal synergies, and disrupted or unbalanced sensory pathways (Hogan et al., 2006). Nevertheless, the most successful form of upper-extremity robotic therapy to date was designed to incorporate principles of motor learning and it has proven effective (Krebs et al., 2003; Miller et al., 2010). It therefore seems probable that something resembling motor learning is at least part of the recovery process.

**Figure 7:**
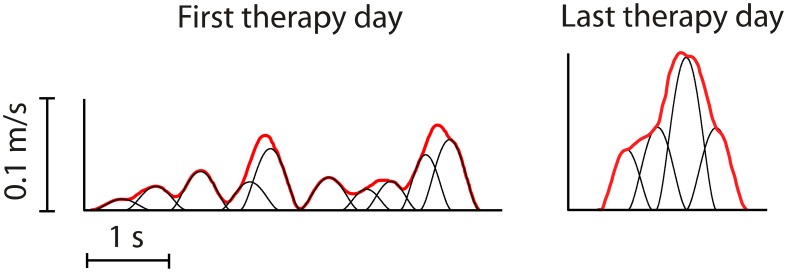
**Typical movement profiles between the same target positions from one patient recorded on the first and last days of therapy (Rohrer et al., 2004)**. Bold lines indicate measured tangential speed. Fine lines indicate submovements identified using a global optimization algorithm. The later profile shows fewer submovements, which have greater peak speed, duration and overlap.

We propose that motor learning (and, by extension, recovery) consists of encoding the parameters of dynamic primitives and subsequently using them to reconstruct the primitives, rather than details of behavior. Support is found in the analysis of infant reaching movements, which initially exhibit submovements but become essentially continuous at around 6 months of age (Hofsten, 1991; Berthier, 1996). More recent work showed that the earliest movements made by patients recovering after a paralyzing stroke were composed of submovements with remarkably stereotyped speed profiles, even for different patients with different lesions (Krebs et al., 1999). This degree of robustness or “temporary permanence” makes a compelling case that submovements are, indeed, a primitive dynamic element of human motor behavior. Studies of movement changes during recovery after stroke (Figure 7) showed that submovements grew progressively larger, fewer, and more blended as recovery progressed (Rohrer et al., 2002, 2004; Dipietro et al., 2009). Whether similar patterns will be found in lower-extremity behavior remains a topic for future research.

Muscular weakness, spasticity and abnormal muscle tone may all manifest as disruptions of mechanical impedance. Because impedance is at the *interface* between the CNS and the physical world, inappropriate impedance may hinder the recovery of effective motor actions. We therefore expect normalization of impedance concurrently with recovery. Support is found in recent preliminary studies of how ankle impedance influences recovery of locomotion. The ankle impedance of neurologically impaired subjects was significantly different from that of age-matched healthy subjects (Roy et al., 2009, 2011; Lee et al., 2011). Robot-aided therapy in which patients were seated with the foot clear of the ground (“open chain”) and performed visually evoked “reaching” movements with the ankle while the robot provided graded assistance as needed successfully resolved the abnormality and—most remarkably—this form of therapy resulted in a 20% improvement of over-ground walking speed (Forrester et al., 2011).

This observation seems to suggest that correcting abnormal impedance due to weakness, spasticity or abnormal muscle tone is a *pre-requisite* for recovery, but caution is appropriate; changing impedance may not be a cause of recovery but a consequence. To elaborate, neurological injury may result in weakened and/or unbalanced descending neural drive from higher levels of the CNS to the periphery. This may alter the excitability of spinal segmental neurons, e.g., increasing reflex feedback gains by reducing inhibition, and that, in turn, may alter impedance. Active participation of the patient is an essential element of the robot-aided therapy that corrected abnormal ankle impedance (Forrester et al., 2011). Active participation may have increased descending drive, leading *both* to more normal impedance and improved overground locomotion. Of course, it must be emphasized that these are mere speculations; further study is needed to test whether they contain any grain of truth.

If motor learning is an essential part of neuro-recovery, we may expect that greater intensity of practice will yield better outcomes, and that is consistent with the success of upper-extremity robot-aided therapy. It might then seem that rhythmic practice should be most effective because it enables a greater intensity of practice—more movements per unit time than discrete movements spanning the same workspace. However, if rhythmic and discrete movements arise from distinct dynamic primitives, then learning one type may not generalize to improved performance of the other. In fact, recent studies of unimpaired subjects' adaptation showed that the benefits of rhythmic practice did not transfer to performance of discrete movements (Ikegami et al., 2010; Howard et al., 2011).

If that result generalizes to lower extremity actions, it might account for some of the difficulties that have thwarted attempts to improve locomotor therapy. Treadmill-based robot-aided therapy has been found less effective than conventional therapy and “… still in its infancy” (Miller et al., 2010). Human-administered locomotor therapy has fared little better: an extensive study of body-weight supported treadmill training found that it yielded no better outcome than a home-based exercise program that was “… expected to have little or no effect on the primary outcome, gait speed” (Duncan et al., 2007, 2011). Both of these treadmill-based approaches emphasized rhythmic practice of walking movements. However, any benefits may have generalized poorly to the wider context of functional walking, which may in addition require discrete actions and controlled impedance. Once again, these are no more than speculations, but they illustrate some of the insight that might be afforded by applying a theoretical framework based on dynamic primitives to both upper- and lower-extremity behavior.

## Future directions

We have attempted to outline how a theory based on dynamic primitives might be applied to describe control of human locomotion as well as object manipulation and the use of tools. This outline is no more than a tentative beginning. Our first concern is that a theory should be *competent* to account for a wide range of observed behavior. Where possible, we have attempted to be faithful to the underlying physiology but that is a secondary consideration at this point; fidelity without competence is useless.

Moreover, complete fidelity is probably unattainable and certainly impractical. For example, functioning nerves and muscles require expression of genes to produce essential proteins but our present knowledge of that process and how it is controlled remains profoundly limited. Even if it were possible, a theoretical description of motor control that attempted to include that level of detail would be hopelessly cumbersome. It would defeat the main purpose of formulating a theory, to gain insight.

Any sufficiently ambitious theory will inevitably be contradicted by some experimental observations but this does not mean that it should be discarded outright. A practical theory should be *incrementally revisable* to accommodate new knowledge as it is gained. Theory-building is an iterative, ongoing process. In order for the revisions to be incremental rather than catastrophic, the foundations must be reliable. This requires the theory—and especially its foundations—to have passed the test of falsification (Ajemian and Hogan, 2010).

That reveals one of the challenges of a theory based on dynamic primitives, and may explain why it has not yet been established despite more than a century of investigation. In order to describe the wide repertoire of human behavior competently, the primitives must exhibit what we have called “composability”—observed behavior may be composed of multiple primitives overlapping in time. But composability also implies that unambiguously identifying primitives solely from measurements at the *observational* level is difficult. If the detailed form of the primitive (submovement, oscillation or impedance) is known, the problem is tractable. Without that knowledge, it is “ill-posed” in the sense that a unique solution may not exist. Some progress has been made on this problem by using optimization to provide regularization, but much remains to be done (Rohrer and Hogan, 2003, 2006).

Of course, scientific studies are not confined to this observational level. Studies at the *physiological* level may resolve the ambiguities. For example, it is not clear whether any convincing evidence of an oscillatory dynamic primitive can be found at the observational level; rhythmic movements could be a combination of back-to-back overlapping submovements in opposite directions. But physiological evidence clearly shows that rhythmic behavior cannot always be dismissed as a combination of submovements and is a distinct dynamic primitive.

Another open question is how many classes of primitives may be required. Here we have considered three—submovements, oscillations and impedances—but there are other possibilities. For example, synergies have been proposed as primitive elements of motor coordination to simplify the problem of managing the many degrees of freedom of the biological motor control system. That may be true, but it is also possible that at least some synergies may be an emergent property of mechanical impedance (Hogan and Sternad, 2012). Which of these possibilities is more competent requires further study.

We expect the parameters of individual exemplars within each class of primitives to be limited but we do not yet know the precise values of those limits. For example, a lower limit to the period of oscillatory movements seems uncontroversial (infinitely rapid movements are physiologically implausible) but there also appears to be an upper limit to the period of primitive oscillatory actions. Beyond that limit, submovements appear to predominate (Doeringer and Hogan, 1998; Dipietro et al., 2004, 2005a,b; van der Wel et al., 2010). Within their limits it is unclear whether the parameters may take on any of a continuous range of values or are confined to a finite set of values. Further research is required.

One essential aspect of our conception of motor control based on primitives is that they are attractors. That prompts the question: which attractors underlie human locomotion? They might be point attractors, e.g., to support foot placement; or trajectory attractors, e.g., to control foot trajectory; or limit-cycle attractors, e.g., to account for the orbital stability of the walking rhythm; or even chaotic attractors as reported by Hausdorff et al. (1995) and Hausdorff et al. (2001). Which of these attractors, or combinations of them, are demonstrable in human locomotion remains to be established.

To conclude, we do not have the temerity to claim that what we have outlined is yet a complete account of upper- and lower-extremity motor behavior. Yet we do contend that such a theory is possible, necessary and timely—perhaps even overdue. Its development will inevitably require considerable hard work from many contributors. To quote Ziman (1969):

This technique, of soliciting many modest contributions to the store of human knowledge, has been the secret of Western science since the seventeenth century, for it achieves a corporate, collective power that is far greater than one individual can exert.

The main thing is to get started. This paper is an attempt to do so.

### Conflict of interest statement

Neville Hogan holds equity in Interactive Motion Technologies, Inc., a Massachusetts corporation that manufactures robotic technology for rehabilitation. The other author declares that the research was conducted in the absence of any commercial or financial relationships that could be construed as a potential conflict of interest.

## Footnotes

^1^To understand the importance of mechanical impedance, consider the difference between kicking a ball and “trapping” it; the former require stiffening the ankle to transfer momentum to the ball; the latter requires relaxing the foot to “deaden” the bounce.
